# Exploring the Impacts of Housing Condition on Migrants’ Mental Health in Nanxiang, Shanghai: A Structural Equation Modelling Approach

**DOI:** 10.3390/ijerph15020225

**Published:** 2018-01-29

**Authors:** Yang Xiao, Siyu Miao, Chinmoy Sarkar, Huizhi Geng, Yi Lu

**Affiliations:** 1 College of Architecture and Urban Planning, Tongji University, 1239 Siping Road, Shanghai 200092, China; yxiao@tongji.edu.cn (Y.X.); msy0207@163.com (S.M.); genghuizhi@163.com (H.G.); 2 Healthy High Density Cities Lab, HKUrbanLab, The University of Hong Kong, Knowles Building, Pokfulam Road, Hong Kong, China; 3 Department of Architecture and Civil Engineering, City University of Hong Kong, Hong Kong, China; yilu24@cityu.edu.hk

**Keywords:** housing condition, neighbourhood satisfaction, mental health, migrants, structural equation modelling (SEM), Shanghai

## Abstract

Although rapid urbanization and associated rural-to-urban migration has brought in enormous economic benefits in Chinese cities, one of the negative externalities include adverse effects upon the migrant workers’ mental health. The links between housing conditions and mental health are well-established in healthy city and community planning scholarship. Nonetheless, there has thusfar been no Chinese study deciphering the links between housing conditions and mental health accounting for macro-level community environments, and no study has previously examined the nature of the relationships in locals and migrants. To overcome this research gap, we hypothesized that housing conditions may have a direct and indirect effects upon mental which may be mediated by neighbourhood satisfaction. We tested this hypothesis with the help of a household survey of 368 adult participants in Nanxiang Town, Shanghai, employing a structural equation modeling approach. Our results point to the differential pathways via which housing conditions effect mental health in locals and migrants. For locals, housing conditions have direct effects on mental health, while as for migrants, housing conditions have indirect effects on mental health, mediated via neighborhood satisfaction. Our findings have significant policy implications on building an inclusive and harmonious society. Upstream-level community interventions in the form of sustainable planning and designing of migrant neighborhoods can promote sense of community, social capital and support, thereby improving mental health and overall mental capital of Chinese cities.

## 1. Introduction

After three decades of reforms and policy of liberalization, China is currently undergoing rapid urban transformation. China’s urbanization rate rose from 17.92% in 1978 to 57.35% in 2016. Nonetheless, the data shows that the urbanization rate of the household population is only about 36%, and there is still a gap relative to the average level achieved in developed countries [1]. Associated with this urbanization phenomenon are large-scale infrastructure projects related to the development of new cities and towns as well as retrofitting of existing ones and a palpable demographic shifts through the influx of rural migrant workers to the cities. The United Nations Development Program (UNDP) projects that China will have an additional 310 million new urban dwellers in the coming 20 years, with the total urban population reaching 1 billion. In other words, one in eight urban residential dwellers on Earth are likely to live in Chinese cities [2]. 

As Chinese urbanism begins to shape its residents’ lives, it brings in several opportunities and challenges to their health and wellbeing [3,4]. Studies in the West have shown that urbanization brings together human capital, innovations, and special political, cultural, economic, and educational opportunities, while it has a negative externalities in the form of health costs at both the psychological and physiological levels [5]. Higher degrees of urbanization have been related with higher the incidences of mental illness [6], and improving mental health and capital of populations and cities is of primary importance [7]. In one of the seminal studies, Bairoch reported significantly higher mortality rates in European cities as compared to adjoining rural areas [8]. Recently, Oculicz-Kozaryn reported quantitative evidence indicating that the largest and densest cities are least happy [9]. Chinese cities are experiencing considerable stress on account of massive rural-to-urban migration and the mental disorders constitute a major burden, accounting for 9.5% of all disability-adjusted life years and 23.6% of all years lived with disability [10,11]. Mental disorders constitute a major health risk for migrants workers [12]. With the prospect of new urban life, migrants are faced with the challenge of establishing themselves in a new social landscape, often quite different from their previous established support networks in terms of sense of belonging and social capital. More importantly, re-settlement, employment, and the pressure to support dependent family members are all associated with a high psychological burden [13] and cause psycho-social stress, anxiety, and depression and other mental disorders [14]. Furthermore, *hukou* or the individual household registration is non-transferable in China, which means that once a migrant moves from rural to urban areas, their entitlements to social and medical benefits are significantly diminished [15], which has negative effects upon quality of life, physical, and mental health. 

Housing is the fundamental component of urban environment lying along the path from urban environment to mental health; high-density congested housing has a negative impacts on mental health, both on account of poor physical environment [16] and due to its potential to interfere with and weaken the family’s social support system [17]. In extreme cases, it can even affect the next generation of children’s psychological health [18]. The World Health Organization (WHO) in 1977 put forward the “everyone is healthy” concept. The WHO in 2009 stressed that healthy cities should be able to encourage, meet, and support the health, well-being, security, social relations, accessibility, mobility, self-esteem, and cultural identity of all social and age groups.

The pathways from housing environment to individual mental health linkages are very complex [19]. In general, the main effects are often mediated by the social environment, physical space, access to social and health services provided by the city, as well as an individual’s state of health, each of which may configure the others [20]. The built environment influences an individual’s lifestyle and health behavior such as diet, physical activity, and active travel and configures the social environment, thereby influencing social interactions and support and hence an individual’s mental health [21]. Therefore, the challenge for Chinese cities (as they evolve) will be to effectively integrate health factors into the urban planning of physical space in the ongoing phase of new urbanization, with an overall objective to enhance mental wealth of cities. 

Studies have shown that housing conditions directly or indirectly affect people’s mental health [22]. Previous research on the impact of housing conditions on mental health, primarily in the Western context, have considered the role of housing typology such as villa, duplex, apartment, and tenureship [23,24,25,26], floor-level [27,28,29], physical conditions [16,30,31,32,33,34], and access to residential facilities [35,36,37,38]. Specifically, Richman found that women living in villas had better mental health than women living in duplex and apartment houses [23]. Also, there are studies that found that residents living in high-rises have poor mental health mainly due to lack of security and social support [39]. Halpern divided 117 low-income families into experimental and reference groups. He reported that improving the housing conditions of the experimental group, such as increasing size of the modern kitchen and bathroom and providing constant hot water resulted in improvements in the mental health of participants of the experimental group, while the mental health of reference groups did not change significantly [40]. 

The housing environment may effect mental via several potential mechanisms.

(1) Amplifying adverse effects of social isolation. Social isolation and the associated lack of social relations may be a first cause of mental disorders [41]. Many studies have found that living in a high-rise leads to social isolation and can even affect a resident’s social relationships and weaken people’s mental health [27,42,43,44].
(2) Facilitating social contact and support. Social support may act as buffers against stress, thereby ameriolating adverse effects on mental health and wellbeing [45,46,47,48]. Through the exploration of the relationship between housing conditions and mental health of 279 housing area shortage residents, Smith found that in terms of the good or middle housing conditions, social support had a mediated effects between housing conditions and mental health, but when the housing conditions are not right, social support cannot alleviate the adverse effects of poor housing conditions on mental health [22].
(3) As a source of physiological and psycho-social stress. The housing micro-environment may directly act as source of stressors [49], and its relationship with mental wellbeing has long been recognized [50,51]. Studies have found associations between elevated levels of psychological distress and stress-related housing conditions including high residential density measured as persons/room [52,53,54] and related unwanted social interactions [55], physical quality of housing [31], and insecurity from crime [56].
(4) Perception and self-assessment has also been know produce effects on an individual’s mental health [57], with low self-assessment being associated with mental health problems such as depression, suicide, irregular diet, and anxiety. High self-assessment has been known to improve mental health by offseting many adverse effects [58].

Prior research, mostly in Western contexts, has also shown that in addition to housing conditions themselves. The macro-level environment within the residential neighbourhood is a key determinant of health [59]. The characteristics of the community itself may also have an impact on individual health. Specifically, access to salutogenic community green space can provide residents with a healthy recreational environment and the opportunity to interact with others, both of which are good for mental health. From the perspective of the environment, good community green space, sanitation conditions, and security encourage more healthy activity behaviors such as exercise, walking, and active travel, which have the potential to improve a resident’s physical and mental health. Hence, ignoring the effects of the macro-level community environment can lead to considerable confoundings [60]. Furthermore, the effects are complex, and community-level environment may interact with individual characteristics [61]. 

**Figure 1:**
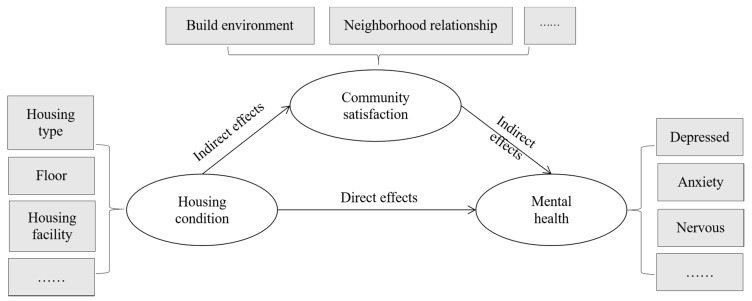
Concept model.

In 2015, the resident population urbanization rate in Shanghai was more than 89%, which is close to the level experienced in most developed countries. Housing conditions for the external migrant population continue to remain a major challenge [62,63]. However, there have thus far been no systematic study on the relationship between housing conditions, the macro-level neighborhood environment, and the mental health of migrants in high density Shanghai. To fill this research gap, we hypothesize that mental health of an individual is a function of micro- and macro-level environments. Direct effects include the effects of housing conditions such as the form of housing type and quality at micro-level directly effecting the trajectory of mental health while, macro-level neighborhood environment configure the level an individual’s neighborhood satisfaction (sense of community) and accrued social capital, indirectly effecting mental health. We further hypothesize that housing conditions may have a direct and/or indirect effects upon mental health, which may be mediated by neighborhood satisfaction, and the mechanisms may be different in the cases of locals and migrants. The present empirical study uses questionnaire survey data derived from the outer area of Shanghai Jiading Nanxiang town. Structural equation modelling (SEM) is a relatively sophisticated (as compared to conventional regression analyses) methodology to model complex effects. We employed SEM to examine the causal pathways between housing conditions, neighborhood environment, and mental health. The conceptual model is illustrated in Figure 1. The objectives of the study are three-fold.

(1) To explore whether housing conditions have a direct effect on mental health;
(2) To examine the effects of housing upon mental health via indirect pathways through neighborhood satisfaction;
(3) To examine potential differences in the effects of housing conditions on mental health (strength and significance of effect estimates) of local and migrant population sub-groups.

## 2. Data and Methods

### 2.1. Study Area and Data Sources

**Figure 2:**
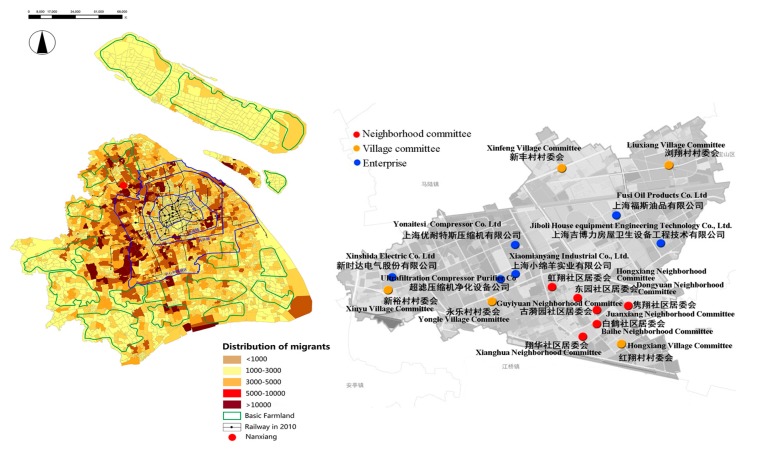
Questionnaire distribution map.

According to the sixth national census data, the city of Shanghai has an area of 6340.50 km^2^ and a total population of 23.02 million, of which the migrant resident population is 8.98 million, amounting to 39%. Most of Shanghai’s new migrants are spatially clustered in the peripheral districts of the city [64]. Therefore, the present study selected participants from these typical areas: Shanghai Jiading Nanxiang Town (Figure 2). A survey was conducted in 2015 employing stratified sampling (STR sampling) and proportional sampling (PPS sampling) for Shanghai local residents and migrants aged 15 or above and living in Nanxiang Town, including the village committee, neighborhood committee, and workplace (enterprise) as the study sampling sites (Figure 2), covering all six neighborhood committees, five village committees, and six major enterprises. In China, the village committee, neighborhood committee, and enterprise are the organizational clusters at the lowest level in rural, urban, and industrial areas. The total number of study participants was 320 (Table 1), with a series of valid questionnaires (293 in total) covering individual socio-demographics, lifestyle, residential environment, and mental health. After processing the missing values, the analytic sample comprised 251 participants, including 114 locals and 137 migrants, the proportion of migrants being 54.6%, which is close to the migrant’s proportion (64.8%) in Nanxiang 2014 [65].

### 2.2. Statistical Method

As mentioned previously, we measured the direct effects of micro-level housing conditions and the indirect effect of the macro-level community environment captured via neighborhood satisfaction based upon mental health indicators. As neighborhood satisfaction is influenced by housing conditions and itself will affect mental health, it can efficiently help to understand the mediated effects. In this paper, the structural equation model (SEM) is used to verify the hypothesis. It is more suitable for dealing with multiple causes and multiple outcomes and involves unobserved latent variables that are indirectly inferred from multiple observed indicator variables [66]. The structural equation model is mainly composed of two parts: the measurement model and the structural model.

(1) $$y=\Lambda_{y}\eta +\varepsilon$$

(2) $$x=\Lambda_{x}\xi +\delta$$

(3) η=Bη+Γξ+ζ

The measurement models (1) and (2) is about the relation between the index and the latent variable, and the structural model (3) imputes the relationship between the latent variables. In this study, the group SEM model was used to study the relationship between housing conditions and mental health of locals and migrants in Shanghai. Housing conditions, neighborhood satisfaction, and mental health were used as latent variables.

### 2.3. Measuring Housing Conditions

Participants’ exposure to specific housing conditions were assessed through a series of questionnaires. Micro-level housing conditions were assessed through the following variables:

(1) Housing typology: This was assessed from participants’ response to the question “Your current housing type is: (a) general building; (b) bungalow; (c) hut; (d) basement; (e) other.” It can be seen from Table 2 that the locals are superior to the migrants regarding the type of housing. Compared with locals, more migrants live in cottage or sheds. Also, the probability of locals and migrants live in a general building is 0.906 and 0.643, respectively. Moreover, for both locals and migrants, the general building is the main type of housing, so to facilitate the model better fit, we divided housing type into “1” and “0” based on whether the housing type is general building or not.
(2) Residential building area in square meters was derived from the participants’ response to the question “What is the size (in square meters) of your residential unit? If you live in a shared household, only estimate the living area of your family.” Residential unit building area was employed as a continuous variable in our models.
(3) Access to basic residential facilities was assessed from a question related to seven residential facility typologies: “Does the house has separate facilities (namely, separate kitchen, separate washroom, shower facilities, access to liquefaction/pipe gas, air conditioning/heating equipment, home balcony, and elevator),” with 1 for a positive and 0 for negative response. Responses to these seven indicators were added as a dummy variable. The CFA test of the nine variables included in the housing condition has a CFI of 0.967 and greater than 0.95, which means the fitting result is good and the latent variables are set up reasonably. These nine indicators can describe the housing quality of locals and migrants.

Neighborhood satisfaction was operationalized from participant responses to questionnaires related to satisfaction with macro-environmental aspects of housing, community facilities, and services. Many studies have shown that access to community facilities and services with residential environments, such as walkable landuses, salutogenic green space, etc., have a beneficial effect on resident’s mental health, in particular for the elderly, women, and those living in low socioeconomic areas [27,36]. In the present study, satisfaction with community facilities and services was employed as a proxy for neighbourhood satisfactions. A set of seven questions were employed to measure satisfaction with various key community facilities: namely, community services, shopping and commercial facilities, community policing, sanitation, recreational facilities, community greening, and property management. Participant responses to these Likert scale questions ranged between 1–5 points (1 = dissatisfied, 2 = dissatisfied, 3 = so-so, 4 = satisfied, 5 = very satisfied). The CFI value of the scale is 0.995, indicating a good fit with the seven indicators acting as a reasonable proxy of neighborhood satisfaction for the locals and migrants.

### 2.4. Outcome: Mental Health

On the topic of mental health, the respondents were asked 12 questions about the extent of their current emotions based on the World Health Organization’s mental health scale. From the 12 indicators, we selected five indicators for this study to measure mental health. The Mental Health Scale is a general instrument for assessing the psychological problems of ordinary people [28], which has been widely used in large sample mental health surveys such as the National Health Survey and the World Health Organization’s World Mental Health Survey (WMH) [29]. The scale contains questions on the frequency of nervousness, despair, anxiety, or upset over the last 30 days. According to the mental health scale and the best fit of our actual data, we selected five indicators as the main indicators of mental health in the present study. These were anxiety and insomnia, nervousness, cannot overcome difficulties, unhappiness and depression, and loss of confidence. Response to each question was based on the frequency of the problem and reported as never, rarely, sometimes, and often, each given 4–1 points. The higher the score, the better the mental health. The reported reliability of the scale was 0.845, and the CFI value of the CFA test was 0.971, indicating a good fit.

## 3. Analysis Results

### 3.1. Sample Characteristics

Table 3 summarizes the residential environment indicators among the 114 locals and the 137 migrants in Nanxiang Town. A non-parametric Kruskal-Wallis test was employed to examine the level of statistical significance of the differences of means across categories. The results of Table 3 indicate that there are significant differences between migrants and locals across indicators of housing conditions, neighborhood satisfaction, mental health, and demographic indicators. Most of the average or quantitative indicators of migrants are lower than those of Shanghai locals. Compared with locals, migrants are exposed to a poorer residential environment. In fact, these poorer housing conditions are reflected in poor housing conditions and in living areas, residential discrimination, and other aspects [30].

Specifically, the differences between the migrants and locals are mainly reflected in the housing conditions. Housing tenureship, quality, and access to facilities were poorer in the migrant category as compared to the locals. Locals mostly resided in self-owned housing (93.7%), while migrants mainly lived in rented accommodation (78.8%). Results indicated that 59.1% of migrants were sole tenants, 19.7% were joint tenants, and the rent of 61.6% tenant was less than 1000 yuan. Morover, locals are significantly more likely to live in commercial housing compared with migrants. The choice of housing for migrants were more biased, with low rent corresponding to increasing commuting distance to place of work. Furthermore, because of the lower socioeconomic status and prominent liquidity characteristics of migrants, their residential environment had a temporary characteristic, resulting in significantly poorer housing quality as compared to non-migrant residents [31]. Also, there was a significant difference in neighborhood satisfaction scores rating community service, property management, and community facilities satisfaction, with migrants scoring significantly lower than Shanghai locals. With respect to mental health, the results of the mental health comparison between residents and foreign population in Nanxiang are similar to those of domestic scholars. There are also some differences in mental health between migrants and locals [32,33,34,35,36]. “Unhappy and depressed” scores for migrants (at the α = 0.05 level) was significantly lower than those of locals, implying there is a higher prevalence of unhappiness and depression among migrants. The mean age of the population also significantly differed between locals and migrants, with the latter’s average age being eight years younger than that of the locals, with participants between 16 to 60 years old accounting for 96.8% of the total population sample. As expected, there were significant differences in income between the local and migrant populations.

### 3.2. Analysis Results

**Figure 3:**
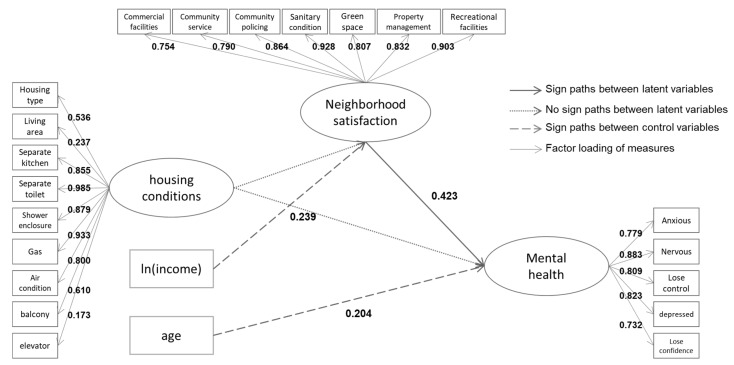
Structural equation model linking housing characteristics to mental health among Shanghai locals.

**Figure 4:**
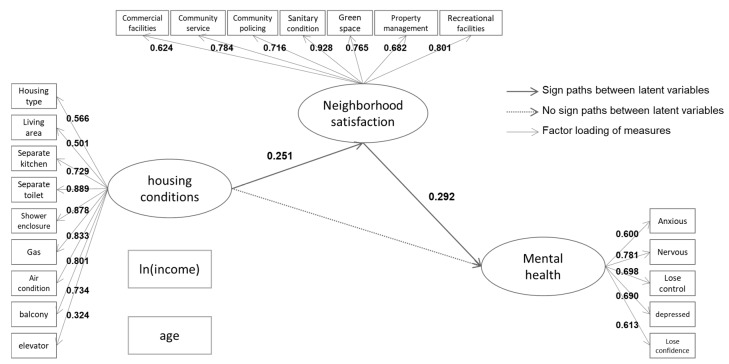
Structural equation model linking housing characteristics to mental health among Shanghai migrants.

Model fit statistics indicate a good fit of the a priori SEM model, with a Chi square test of model fit at 844.50 on 486 degrees of freedom and RMSEA of 0.084. The model fitting index was close to one—CFI = 0.880 and TLI = 0.875. The structural equations model illustrated in Figure 3 and Figure 4 examines the impact of housing conditions and neighborhood satisfaction on mental health among Shanghai locals and migrants, respectively. The study reported differences in the mechanism by which housing conditions affect mental health among locals and migrants.

For Shanghai locals (Figure 3), the path from housing conditions to neighborhood satisfaction was not significant. It was found that the direct effect of housing condition on local participants’ mental health was not significant, at 95% confidence level (*p* = 0.055), and borderline significance (*p* < 0.10) indicated the mild protective effects of better housing conditions upon mental health. As per expectations, the path coefficient of neighborhood satisfaction upon the mental health was 0.423 (*p* = 0.000), pointing to accrued beneficial effects upon mental health. Moreover, the older the person is, the better his mental health will be, while age had no significant effect on neighborhood satisfaction. Income was observed to directly effect locals’ neighborhood satisfaction but did not influence mental health.

On the contrary, in the case of migrants (Figure 4), housing conditions per se had no significant direct effect upon mental health (*p* = 0.187), the effects were mediated via neighborhood satisfaction. Housing conditions were linked with neighborhood satisfaction with a path coefficient of 0.251 (*p* = 0.013), and the direct path coefficient from neighborhood satisfaction to mental health was 0.292 (*p* = 0.006), which may imply that although the housing conditions of migrants do not directly affect mental health, improving the housing conditions of migrants constitutes a better perception of neighborhood, and increments in neighborhood satisfaction have a significant positive impact on mental health. The effects of age and income on community satisfaction and mental health of migrants remained insignificant.

The results are summarized in Table 4. In a nutshell, in the case of migrant participants. housing conditions had an indirect effect upon mental health, the effect being mediated by neighborhood satisfaction. For local participants, housing conditions were not linked with neighborhood satisfaction, while income had indirect effect on mental helath via neighborhood satisfaction. 

## 4. Discussion

In the present study, we examined the effects of housing conditions upon mental health among participants from Nanxiang Town in Shanghai through a structural equation modelling approach. The application of SEM enabled isolation of both direct and indirect pathways along which housing condition influences on people’s mental health. Specifically, our results showed that housing conditions have indirect effects on migrant’s mental health via neighborhood satisfaction, while for local participants, housing conditions posed a direct effect on mental health.

Our study provides substantial evidence that housing conditions in China act along two distinct causal pathways to produce effects upon individual’s mental health [22]. Neighborhood satisfaction was reported to mediate the effect of housing conditions on the mental health of Shanghai migrants. The results compliment prior evidence that showed that neighborhood effect does matter for social integration and cohesion issues in Shanghai [67,68]. They point to the fact that a community with greater satisfaction can provide a sense of belonging to its inhabitants, and enhanced social support can mitigate the psychological pressure derived from poor housing conditions [12,39]. 

The principle finding that two specific mechanisms exist in the case of local and migrant populations, respectively, where housing conditions effect mental health is of significant relevance. In principle, this points to the underlying causal pathways. Local residents are associated with residential stability and, as our data shows, have access to better quality housing and neighborhood environments. Housing conditions did not directly or indirectly produce significant effects on mental health, while neighborhood satisfaction directly influenced mental health. The relationship between income and mental health was also mediated via neighborhood satisfaction. However, in the case of migrants, housing conditions did not directly affect mental health but had significant indirect effects mediated via neighborhood satisfaction. Migrants are more likely to self-select housing; the choice is dependent mostly on economic considerations of affordability such as residential rent instead of housing quality per se [69]. The detected relationship between housing conditions and neighborhood satisfaction in migrants is indicative of the socio-spatial clustering of poor migrant housing in deprived neighborhoods as experienced in Shanghai. The null direct effects of housing conditions may be explained on the basis of lack of affordability, a sort of psychological saturation wherein there is no expectation among the migrants of better quality housing. It may also be related to the actual time spent in residences. Often, migrant workers do multiple jobs, spending longer hours outside for sustenance with significantly less time spent within the residence. The observed indirect effects of housing conditions mediated by neighborhood satisfaction and the direct effects of the later upon mental health may be a result of a number of underlying factors. First, migrants are inherently associated with broken social network [5] when they migrate to cities, since they generally do not bring relatives with them, their children still stay in their hometown [28], and hence migrants have weak social tie support. Second, poorer economic conditions imply they are segregated to living in deprived neighborhoods with a reduced sense of community and safety (higher fear of crime). Third, unstable job contracts mean lower residential stability and higher rate of migration between neighborhoods with generally similar housing conditions. In light of these factors, a healthy community environment—one that promotes sense of community and social support and is associated with enhanced perception of territoriality, defensible space, and safety—is of critical importance in mental wellbeing. In contrast, housing conditions, community environment, and community services are the primary consideration in local people’s choices regarding accommodations. We acknowledge the cross-sectional design and small scale as limitations of the present study. Largescale studies involving more townships with longitudinal design are needed to further validate these findings. Such largescale studies with significant heterogeneity in population characteristics must also adjust for differences in lifestyles arising from inherent cultural affinities.

## 5. Conclusions

Mental wealth constitutes an indispensable resource of any sustainable global city. In a rapidly urbanizing city such as Shanghai, the mental health of the migrant workforce is one of the primary considerations in the economics of developmental pressures versus socio-spatial and health disparities. Our findings have significant policy implications, especially at the level of community health planning and management. As an important upstream-level community intervention to minimize health disparities, it is important to optimize the density and mix of local-to-migrant housing to promote a sense of community and enhance people’s social capital and wellbeing [69]. Provisioning access to key community services is also significant; for instance, with the evidence confirming the environment injustice and inefficient access to public parks in Shanghai [70,71,72,73], the local government should aim to improve the quality of green services to provide a livable and inclusive environment promoting mental health of the migrant workers and hence enhancing the overall mental capital of the city. At an institutional level, policy makers have a role to further refine policies related to *hukou* registration with a view to improve access to basic services (reduce their disparities) that adversely affect migrant workers’ mental health.

As Chinese cities continue to rapidly urbanize, planning sustainable housing provisions for migrant workers will ensure social sustainability via reducing disparity and accrue significant economic benefits through reductions in chronic disease and mental illnesses among vulnerable migrants and related costs of treatment and care in the long run. 

## Figures and Tables

**Table 1:** Distribution of study participants.

Neighborhood Committee	Number	Village Committee	Number	Enterprise	Number
Guyiyuan	7	Xinyu	27	Giboli	17
Dongyuan	6	Yongle	48	Xinshida	28
Juanxiang	9	Hongxiang	16	Fuxiyoupin	21
Baihe	8	Xinfeng	8	Xiaomianyang	22
Xianghua	11	Liuxiang	23	Chaolv	22
Hongxiang	8	/	/	Younaitesi	39
Total of neighborhood committee questionnaires	49	Total of village committee questionnaires	122	Total of enterprise questionnaires	149
Total	320				

**Table 2:** Housing type among the local and migrant categories.

Housing Type	Local	Migrant
general building	108	101
Bungalow	2	26
Hut	0	6
Basement	0	0
Other	7	24
Total	117	157

**Table 3:** Descriptive characteristics of the participants’ residential environment.

Variables	Local (*n* = 114)	Migrant (*n* = 137)	*p*-Value of Difference
Housing conditions			
Commercial residential building; *N* (%)	108.00 (94.7%)	101.00 (73.7%)	***
housing area; Mean (SD)	53.70 (30.77)	47.10 (24.14)	*
Facilities; *N* (%)			
Separate kitchen	106.00 (93.0%)	93.00 (67.9%)	***
Separate toilet	105.00 (92.1%)	87.00 (63.5%)	***
Shower	105.00 (92.1%)	96.00 (70.1%)	***
Gas	104.00 (91.2%)	82.00 (59.9%)	***
Air condition	103.00 (90.4%)	95.00 (69.3%)	***
Balcony	96.00 (84.2%)	69.00 (50.4%)	***
Elevator	39.00 (34.2%)	37.00 (27.0%)	
Neighborhood satisfaction; Mean (SD)			
Community services	3.67 (0.88)	3.20 (0.92)	***
Commercial facilities	4.35 (0.96)	4.15 (1.15)	
Community policing	3.60 (0.90)	3.50 (0.92)	
Sanitary condition	3.53 (0.92)	3.34 (0.93)	
Recreational facilities	3.32 (0.99)	3.13 (0.99)	
Green space	3.50 (1.01)	3.31 (0.95)	
Property management	4.36 (0.96)	4.10 (0.99)	*
Mental health; Mean (SD)			
Insomnia because of anxiety	2.82 (0.90)	2.71 (0.88)	
Always feel nervous	1.97 (1.19)	1.76 (1.09)	
Cannot overcome difficulties	2.68 (0.80)	2.61 (0.79)	
Be unhappy and depressed	2.83 (0.89)	2.66 (0.79)	*
Lose confidence	3.01 (0.92)	3.07 (0.82)	
Age; Mean (SD)	40.21 (13.14)	31.83 (7.22)	***
Gender; *N* (%) (female as reference)	58.00 (50.9%)	73.00 (53.3%)	
Income (ln); Mean (SD)	1.50 (0.67)	1.75 (0.51)	**

* *p* < 0.05; ** *p* < 0.01; *** *p* < 0.001 two-tailed *t*-tests chi-square test.

**Table 4:** Results of impact analysis.

Variable	Neighborhood Satisfaction		Mental Health					
			Direct Effect		Indirect Effect		Total Effect	
Local								
Housing condition	0.54		0.60		0.20		0.80	**
Age	−0.00		0.01	*	−0.00		0.01	*
Income(ln)	0.28	*	−0.10		0.11	*	0.00	
Migrants								
Housing condition	0.48	*	−0.23		0.11		−0.11	
Age	0.01		0.01		0.00		0.01	*
Income(ln)	0.01		−0.11		0.00		−0.11	

* *p* < 0.05; ** *p* < 0.01; *N* = 251.
